# Recurrent Catatonia in the Setting of Urinary Tract Infection and Medical Comorbidity: A Case Report

**DOI:** 10.7759/cureus.113615

**Published:** 2026-07-29

**Authors:** Paula Traugott, Rachel Mimbella, Jessica C Irizarry Flores, Helen H Kyomen

**Affiliations:** 1 Psychiatry, Boston Medical Center-Brighton, Boston, USA; 2 Psychiatry, Boston University Chobanian and Avedisian School of Medicine, Boston, USA; 3 Psychiatry, Tufts University School of Medicine, Boston, USA; 4 Psychiatry, Harvard Medical School, Boston, USA

**Keywords:** benzodiazepines, catatonia, electroconvulsive therapy, immune dysregulation, nmda antagonists, urinary tract infection, zolpidem

## Abstract

Catatonia is a neuropsychiatric syndrome characterized by disturbances in motor activity, affect, and autonomic function. Although often associated with primary mood or psychotic disorders, it can be precipitated or sustained by systemic infections such as urinary tract infection (UTI). We describe a 60-year-old man with recurrent catatonia and diagnoses of schizophrenia, bipolar disorder, and major neurocognitive disorder, whose latest episode of catatonia, described in this case report, was preceded by psychosocial stress and appeared to be exacerbated by polymicrobial UTI, bacteremia, and urosepsis. He had a history of neuroleptic malignant syndrome (NMS) and an inconsistent response to electroconvulsive therapy (ECT), limiting usual first-line treatment options. During this admission, he developed *Klebsiella pneumoniae* UTI with *Staphylococcus simulans* bacteremia and later *Pseudomonas aeruginosa* urosepsis, in the context of mixed central and nephrogenic diabetes insipidus likely related to long-term lithium therapy. The patient’s catatonia proved refractory to very high-dose lorazepam but improved with an intensive, multimodal regimen including intravenous (IV) benzodiazepines (diazepam, midazolam), enteral and subsequently oral lorazepam, zolpidem, memantine, and aggressive treatment of infection and electrolyte abnormalities. He ultimately regained baseline function and was discharged on a slow taper of benzodiazepines and zolpidem. This case illustrates the bidirectional relationship between infection, autonomic and immune dysregulation, and catatonia; highlights practical challenges when treatment with benzodiazepines and ECT is constrained by medical comorbidity; and supports considering adjunctive GABAergic and glutamatergic agents in carefully selected cases of refractory catatonia, under close specialist supervision.

## Introduction

Catatonia is a severe neuropsychiatric syndrome characterized by alterations in motor activity, affect, and autonomic function [1-3]. While it is most associated with psychiatric conditions such as mood and psychotic disorders, catatonia can also occur secondary to neurologic or medical conditions, including infections [4-6]. Its complex and incompletely understood pathophysiology is thought to involve dysregulation of GABAergic, dopaminergic, glutamatergic, and serotonergic systems, with emerging evidence suggesting a role for immune-inflammatory mechanisms [5,7-12]. Catatonia presents with a heterogeneous clinical picture, and its diagnosis relies primarily on observable signs, sometimes supported by formal rating scales such as the Bush-Francis Catatonia Rating Scale (BFCRS) [8,12,13]. Prompt recognition is critical, as untreated catatonia can lead to serious medical complications, including dehydration, malnutrition, deep vein thrombosis, pulmonary embolism, and infections, among others [1,8]. First-line management of catatonia typically includes benzodiazepines and electroconvulsive therapy (ECT), with alternative pharmacologic and neuromodulation strategies considered in refractory cases [1,9,12,14]. Optimal treatment requires an individualized, multidisciplinary approach that addresses both psychiatric stabilization and medical safety. This case report describes the clinical course and management of a patient with recurrent, treatment-resistant catatonia in the context of complex medical comorbidities, particularly recurrent urinary tract infection (UTI). This case is particularly instructive given the patient's history of neuroleptic malignant syndrome (NMS) limiting antipsychotic use, inconsistent ECT response, and lithium-associated diabetes insipidus, all of which constrained standard therapeutic options and necessitated ICU-level treatment. It aims to illustrate the temporal association between infection, potential immune system dysregulation, and catatonic relapse, and to highlight practical therapeutic considerations for individualized, multimodal treatment in medically complex, refractory catatonia.

## Case presentation

This case describes a 60-year-old man with a longstanding psychiatric history of bipolar disorder, schizophrenia, recurrent catatonia, and dementia with behavioral disturbance, who was admitted to a hospital in Boston, Massachusetts, from his skilled nursing facility after a psychosocial stressor, the recent breakup with his girlfriend, preceded a relapse of catatonia, which was characterized by mutism, stupor, negativism, and non-responsiveness. For the past eight years, he has experienced recurrent catatonic episodes, frequently in the setting of medical illness, particularly UTIs, which appeared to precipitate or exacerbate neuropsychiatric decline.

The patient's psychiatric illness began at age 17 with auditory hallucinations and paranoid ideation. He has a history of seasonal mania, typically in the spring, and a family history of psychotic disorders affecting one of his brothers. Approximately nine years ago, he began experiencing recurrent episodes of catatonia, often in the context of medical illness, particularly urinary tract infections and urosepsis, requiring multiple inpatient hospitalizations. His catatonic episodes characteristically present with profound alterations in speech and swallowing, jaw clenching or bruxism, and loss of facial motor control.

**Figure 1:**
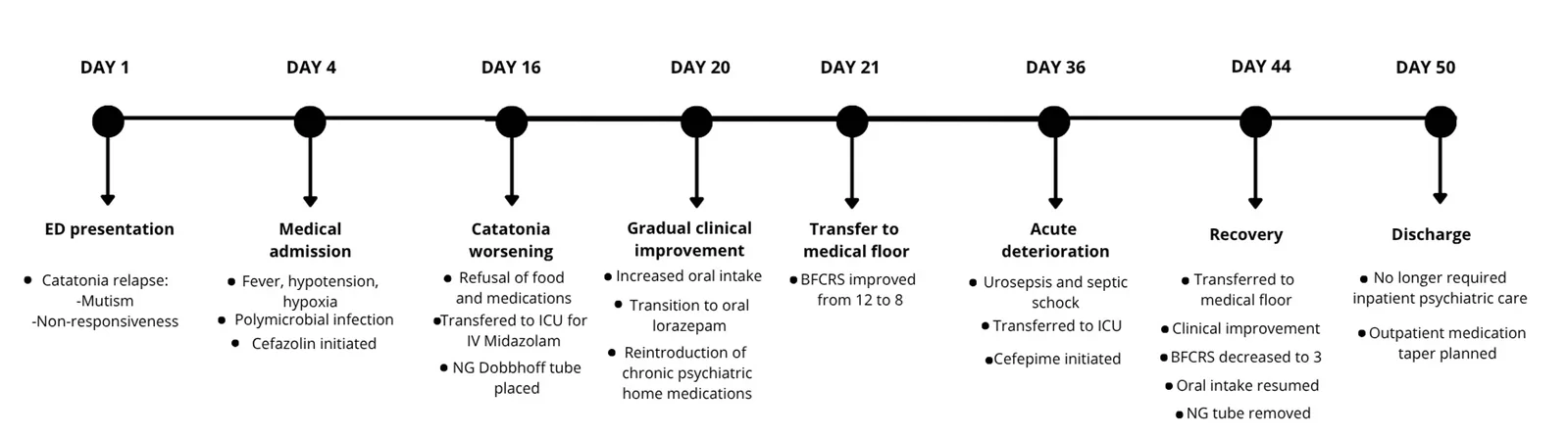
Chronological summary of the patient’s clinical course ED: emergency department, ICU: intensive care unit, IV: intravenous, NG: nasogastric, BFCRS: Bush-Francis Catatonia Rating Scale

Management of his catatonia has been particularly challenging due to the following considerations: (1) a prior episode of NMS related to antipsychotic treatment given for his underlying schizophrenia; and (2) ECT producing inconsistent results (i.e., his most recent ECT course led to neurologic decline). During his last major hospitalization in 2024, he recovered after four months of high-dose benzodiazepines (intravenous (IV) lorazepam up to 30 mg/day), zolpidem (up to 40 mg/day), and memantine (up to 40 mg/day). Upon discharge, his condition remained psychiatrically stable on a regimen that included zolpidem, memantine, clonazepam, lithium, and valproic acid until he relapsed in 2025, which led to the episode described in this report. Compared with his prior hospitalization, this episode ultimately required a similarly high overall benzodiazepine burden, now delivered through a combination of IV diazepam (up to 15 mg/day), IV midazolam (up to 8 mg/day), and enteral and subsequently oral lorazepam (up to 12 mg /day). His full hospital course is summarized in Figure 1.

On the third day of this admission for catatonia, the patient developed fever, hypotension, and hypoxia. Microbiologic workup revealed polymicrobial infection: *Klebsiella pneumoniae* in the urine (UTI versus prostatitis) and *Staphylococcus simulans* bacteremia associated with an infected port (likely from prior treatments requiring IV access, such as ECT). The port was removed, and cefazolin was initiated via a peripherally inserted central catheter (PICC) for four weeks as definitive therapy for both organisms. Despite infection control, his catatonia worsened, with refusal of oral medications and food. Oral lorazepam was eventually transitioned to IV diazepam due to a shortage of intramuscular (IM)/IV lorazepam in Massachusetts. Following a 3 mg IV dose and a visit from a nurse from his facility, the patient briefly showed marked improvement: he ate his full lunch and expressed interest in walking with physical therapy. However, this was a transient response, and the following days were marked by progressive catatonic features, including decreased oral intake, reduced engagement, and medication refusal. Consequently, the team initiated IV midazolam, necessitating transfer to the intensive care unit (ICU) for administration of this critical care medication. 

While in the ICU, the patient received midazolam up to 2 mg four times daily and was started on enteral nutrition via nasogastric (NG) Dobhoff tube. Over the following days, he demonstrated gradual improvement, regaining the ability to follow commands and showing intermittent volitional behavior. During this period, the patient's persistent hypernatremia prompted nephrology and endocrinology consultation. Evaluation revealed mixed central and nephrogenic diabetes insipidus, likely secondary to chronic lithium use. The patient was treated with amiloride and desmopressin, leading to partial correction of sodium levels. Recognition and partial correction of his mixed central and nephrogenic diabetes insipidus were crucial to safe benzodiazepine titration, as persistent hypernatremia and volume depletion risked further neurologic compromise. As his enteral tolerance improved, he was transitioned from midazolam to oral lorazepam. Zolpidem was reintroduced (up to 40 mg/day), resulting in further improvement of catatonic symptoms. In addition to benzodiazepines and zolpidem, his chronic psychiatric medications (valproic acid, memantine, lithium, ramelteon, mirtazapine, and melatonin) were reintroduced and maintained through his NG tube.

After one week in the ICU, the patient was transferred back to the medical floor. Under escalating doses of lorazepam and zolpidem, his catatonia continued to improve, with his BFCRS score decreasing from 12 to 8 [8,12-13]. He became more interactive, followed simple commands, and initiated brief verbal communication. However, after nearly two weeks of relative hemodynamic stability, the patient experienced an acute decline with fever and respiratory distress. Blood cultures grew *Pseudomonas aeruginosa*, consistent with urosepsis and septic shock. Following treatment with cefepime and stabilization again in the ICU, his mental and motor functions improved once again.

In the subsequent weeks, the patient showed steady recovery. His BFCRS score decreased to 3, and he was alert, cooperative, and communicative. He resumed oral intake, the NG tube was removed, he participated in physical therapy, and he denied psychotic or mood symptoms. Psychiatry determined that he no longer met criteria for inpatient psychiatric care and recommended a gradual outpatient taper of benzodiazepines and zolpidem.

## Discussion

Diagnosis and clinical features

Catatonia is a complex and potentially life-threatening neuropsychiatric syndrome characterized by prominent and sometimes conflicting disturbances in motor tone, behavior, and affect, and may be accompanied by autonomic dysfunction [1-3]. Our patient illustrates several increasingly recognized dimensions of catatonia: vulnerability to relapse in the context of systemic infection (particularly recurrent UTI and urosepsis), the contribution of chronic medical comorbidities such as lithium-induced diabetes insipidus, and the challenges of managing treatment-resistant catatonia when antipsychotics and ECT are constrained by prior neuroleptic malignant syndrome and cognitive decline.

According to the Diagnostic and Statistical Manual of Mental Disorders, Fifth Edition (DSM-5), a diagnosis of catatonia requires the presence of at least three of the following features: stupor, mutism, catalepsy, waxy flexibility, negativism, posturing, mannerism, stereotypy, agitation not influenced by external stimuli, grimacing, echolalia, or echopraxia [4,8,15]. In terms of clinical phenotype, catatonia is frequently described using three motor-based subtypes: hypokinetic (retarded or stuporous), which involves immobility, rigidity, and negativism; hyperkinetic (excited), marked by prolonged psychomotor agitation; and parakinetic, defined by abnormal, purposeless, and contextually inappropriate movements [1,8].

Unlike many psychiatric disorders that are primarily diagnosed through subjective symptoms, catatonia is predominantly assessed through observable or elicitable signs [12], making formal rating instruments such as the BFCRS particularly valuable for detection and monitoring [8,13]. Nevertheless, while rating scales serve as valuable tools for systematizing assessment, the formal diagnosis of catatonia should still rely on established diagnostic criteria, such as those outlined in the DSM-5 [1,4]. Although not officially listed as a diagnostic criterion in the DSM-5, a favorable response to a lorazepam challenge (reflected by a 50% reduction in symptoms on a standardized rating scale) can enhance diagnostic confidence in identifying catatonia [1,12].

Medical triggers

Catatonia is not solely a psychiatric condition; rather, it can be triggered by a diverse range of medical disorders, highlighting the importance of thorough medical evaluation and the critical interaction between physical and mental health [5-6]. In our patient, recurrent UTIs and urosepsis appeared temporally linked to catatonic exacerbations, consistent with a para-infectious encephalopathy model, defined as a global neurological decline linked to infection, in which systemic infection and inflammatory cytokines modulate dopaminergic and glutamatergic pathways. UTIs are common comorbidities in neuropsychiatric settings and may not only trigger but also worsen existing neuropsychiatric symptoms. This worsening, particularly in patients with preexisting psychiatric or neurological conditions, has been described within the para-infectious encephalopathy framework. Several mechanisms have been proposed to explain the association of UTI and neuropsychiatric symptoms such as catatonia. Neuropsychiatric illnesses may lead to behavioral or physiological changes such as poor hygiene or urinary retention from medications like antipsychotics, which increase susceptibility to UTIs. Additionally, UTIs may trigger or exacerbate psychiatric symptoms via immune system activation. Inflammatory cytokines, especially interferon-gamma, can alter neurotransmission through dopaminergic and glutamatergic pathways, potentially contributing to neuropsychiatric disorders or neurodegeneration. Chronic infection may also trigger autoimmune processes through molecular mimicry, further damaging neural tissue. These findings point to the need for increased awareness and investigation into immune-inflammatory markers and their role in neuropsychiatric presentations associated with UTIs [6].

Growing evidence suggests that immune system dysregulation plays a key role in the pathophysiology of neuropsychiatric disorders, involving both the innate and adaptive immune systems. This concept extends to catatonia, where the brain’s response to inflammation may result in complex motor disturbances. Furthermore, psychological stress and infection can trigger pro-inflammatory cytokine release, inducing a hypoactive motor state that may become maladaptive and cause further dysfunction. In fact, immobilization itself may activate the innate immune system, potentially contributing to a vicious cycle of sustained immune activation [5]. From a laboratory perspective, certain biomarkers and immunological abnormalities have been associated with catatonia. Moreover, a broad range of autoantibodies have been identified in association with catatonia, including NMDA receptor antibodies, ANA, anti-dsDNA, GABA-A receptor antibodies, and others, suggesting that autoimmune disorders may underlie some presentations of catatonia [12]. Though conclusive evidence linking immune activation directly to catatonia is still limited, this underscores the importance of further exploring the immune-catatonia connection for future diagnostic and therapeutic advances [5,12].

Pathophysiology

Catatonia’s complex pathophysiology is also believed to involve disruptions in motor-affective neural circuits, contributions from inflammation and oxidative stress, and dysregulation of several neurotransmitters [11]. Current evidence suggests that GABA-A receptor hypoactivity, dopamine D2 receptor hypoactivity, glutamatergic NMDA receptor hyperactivity, and alterations in serotonin (specifically, increased 5-HT1A receptor activity) play important roles in catatonia initiation and progression [7-11]. These neurotransmitter systems exert dynamic and interdependent influences on brain circuits involved in catatonic behavior, such that disruption in any one system can alter the overall neurochemical balance and either trigger or alleviate catatonic states. The "universal field hypothesis" posits that catatonia arises from the complex interplay between multiple neurochemical systems rather than from isolated dysfunctions, reinforcing the idea of a multifactorial and interconnected neurobiological foundation for the syndrome [7].

The GABA hypothesis of catatonia is largely supported by the clinical response to lorazepam, a benzodiazepine that acts as a positive allosteric modulator at GABA-A receptors [5,7]. More recently, this hypothesis has evolved into a distinction between GABA-A and GABA-B receptor involvement. GABA-A receptor activation appears to ameliorate catatonia, as shown by the successful use of zolpidem (a non-benzodiazepine GABA-A agonist) in some patients. Conversely, GABA-B receptor activation, such as with baclofen, has been linked to catatonia onset in case reports, suggesting that an imbalance between GABA-A and GABA-B receptor activity may be involved [7]. In addition to its role in the central nervous system, GABA-A contributes to autonomic regulation of micturition by facilitating parasympathetic-mediated detrusor contraction, thereby promoting urination [16]. Moreover, it has been hypothesized that dysfunction in GABAergic pathways may also disrupt sphincter control and voiding reflexes [16-17]. Together, these findings suggest that GABA-A hypoactivity may play a central role in the motor, affective, autonomic, and possibly immunological features of catatonia, although individual variation in receptor subtypes and associated pathways likely contributes to the heterogeneity in presentation and treatment response [5,7,11,16-17].

The dopamine deficiency hypothesis suggests that reduced dopaminergic activity, especially through D2 receptor blockade, may precipitate catatonic symptoms [7]. Therapeutically, dopamine agonists and precursors may help alleviate catatonic symptoms by enhancing dopamine transmission and promoting the striosomal direct pathway. This could facilitate thalamic activation and increase activity in motor-related cortical regions, such as the supplementary and primary motor cortices (a mechanism also observed in akinetic mutism) [12]. In line with this, amantadine, a drug with both dopamine-releasing and agonist properties, has shown clinical efficacy in some cases of catatonia, supporting its role in modulating dopaminergic pathways [7].

The glutamate hypothesis of catatonia centers on dysfunction in the NMDA receptor system, a key component of glutamatergic neurotransmission [7,16]. Additional support comes from the reported efficacy of amantadine and memantine (noncompetitive NMDA antagonists) in selected cases of catatonia, particularly those resistant to standard treatments like lorazepam or ECT. These agents likely act through complex mechanisms involving glutamate modulation, providing indirect evidence for the potential role of NMDA antagonism in catatonia management [10]. While alterations in glutamatergic signaling, especially at NMDA receptors, have been implicated in catatonia, the strength of the evidence remains limited, and further research is necessary to clarify glutamate’s role in the disorder [10,16].

Activation of 5-HT1A serotonin receptors is thought to contribute to catatonia, and agonism at these receptors may play a key role in its pathogenesis. Given the complexity of the serotonin system, which involves multiple receptor subtypes, further research is needed to clarify their specific involvement in catatonia [7].

Treatment

The first-line treatment for catatonia typically depends on the subtype and clinical urgency (which is also influenced by the availability of treatment options), with benzodiazepines and ECT being the most widely used and evidence-supported options [9,12,14]. Despite their frequent use, earlier literature noted a lack of standardized treatment guidelines and no uniform protocol for catatonia management, emphasizing the need for further high-quality intervention studies [9]. Recognizing these gaps, several national and international psychiatric organizations developed guidelines addressing catatonia management, recommending benzodiazepines, glutamate antagonists (e.g., amantadine and memantine), zolpidem, or ECT [8,12]. Taken together, these observations highlight a significant gap in the literature for a comprehensive, multidisciplinary consensus guideline that thoroughly evaluates existing evidence and provides clear treatment recommendations [12].

Benzodiazepines, especially lorazepam, are widely regarded as the first-line treatment for catatonia, offering rapid and often dramatic symptom relief within hours to a few days of administration [9,18]. These medications act as positive allosteric modulators of GABA-A receptors, bolstering impaired GABAergic signaling believed to underlie catatonia [1,16]. Clinical use generally follows a three-phase structure: the challenge test, followed by an acute treatment phase, and then maintenance [1]. Dosing varies widely and may be administered orally, intramuscularly, or intravenously, depending on the patient’s condition [9]. While benzodiazepines are usually well tolerated, approximately 27% of catatonic patients may not respond to lorazepam alone [11]. Low serum iron has been identified as a predictor of benzodiazepine nonresponse in catatonia, underscoring the potential role of biological factors in treatment resistance [18]. Despite its prevalence in treatment, there is no evidence indicating a superior benzodiazepine agent, administration route, or duration; moreover, optimal tapering strategies remain undefined [1,9]. In cases of chronic or recurrent catatonia, maintenance therapy is advised, ideally tapered slowly, to reduce the risk of relapse [1].

When benzodiazepines offer limited benefit, in cases of severe or malignant catatonia, in life-threatening catatonic conditions (including severe dehydration, autonomic dysregulation, thrombosis), and in chronic catatonia defined as symptoms persisting for tens of days, ECT proves particularly valuable, and the general recommendation is to initiate it promptly [1,9]. ECT is highly effective, with response rates ranging from 80% to 100%, including up to 60% of patients who do not improve with benzodiazepines alone [1,16]. Many patients also benefit from concomitant maintenance benzodiazepine therapy alongside ECT [1]. Typically, bilateral ECT is used, with a treatment frequency most often set at three times weekly and session counts varying from 3 to 35 [12]. While the precise mechanism remains unclear, ECT’s effectiveness may partly derive from its capacity to enhance GABA neurotransmission, aligning with the broader GABA hypothesis in neuropsychiatry [16]. Interestingly, ECT also appears to modulate immune function: a single session may temporarily increase cytokines such as IL‑1β, IL‑6, IL‑10, and TNF‑α, whereas multiple sessions may ultimately reduce immune activity [5]. The introduction of ECT has contributed to a decline in catatonia-related mortality, but despite its effectiveness, it remains underutilized due to limited access [11,12,16].

While most patients respond well to either benzodiazepines or ECT, a subset continues to experience only partial or no improvement [12,16]. When these first-line treatments are unavailable, ineffective, or only partially effective, several alternative management strategies (mostly supported by case reports, small series, or expert consensus) may be considered [1,12]. These include NMDA receptor antagonists (e.g., amantadine, memantine), anticholinergics (e.g., benztropine, trihexyphenidyl), antiseizure medications (e.g., carbamazepine, valproate, topiramate), second-generation antipsychotics (SGAs), zolpidem, dopaminergic agents (e.g., carbidopa-levodopa), and neuromodulation techniques (e.g., transcranial magnetic stimulation (TMS) and transcranial direct current stimulation (tDCS)) [1]. Together, these options underscore the need for flexible, multimodal approaches when standard therapies are insufficient, while reinforcing the urgency of further research to establish clearer efficacy data.

Given increasing evidence of glutamatergic involvement in catatonia, NMDA antagonists like amantadine and memantine have emerged as second-line options for catatonia through both direct glutamate antagonism and indirect modulation of GABA and dopamine systems [1,10,12]. These agents may be used as adjuncts to benzodiazepines or, in some cases, as monotherapy. Given their mechanism, they are particularly appropriate for patients with schizophrenia-associated catatonia, which often shows reduced response to benzodiazepines [1]. Both drugs act as uncompetitive NMDA receptor antagonists and are thought to down-regulate glutamatergic hyperactivity, a proposed contributor to catatonia, by restoring balance within cortico-striato-thalamo-cortical (CSTC) circuits. Specifically, their mechanism involves modulation of GABA-A parvalbumin interneurons in the prefrontal cortex and reduction of NMDA activity in the striatum, thereby indirectly compensating for GABA and dopamine deficits [10]. Amantadine also possesses anticholinergic activity, which, together with its dopaminergic effect, has been associated with a potential reduction in NMS-related mortality [18]. Furthermore, amantadine has shown benefit in lorazepam-refractory catatonia, with some experts recommending its trial prior to ECT initiation. Memantine, a structural derivative of amantadine, shares similar NMDA receptor antagonism but lacks dopaminergic activity, which may reduce the risk of psychosis compared to amantadine, making it a suitable alternative when amantadine response is inadequate or side effects develop. Other agents, such as minocycline, have also been used anecdotally when traditional agents fail. In two reported cases of catatonic stupor in schizophrenia, adjunctive minocycline led to clinical improvement, possibly via NMDA antagonism, although its central nervous system (CNS) mechanisms are not well understood [10]. Our patient’s partial response to memantine during both hospitalizations is consistent with reports of benefit from NMDA antagonists in lorazepam-refractory catatonia, particularly in schizophrenia-associated presentations.

Anticholinergic agents and certain anticonvulsants have also been employed as adjunctive therapies, although supporting evidence is limited. Antiseizure medications, including valproate, carbamazepine, and topiramate, have shown potential benefit in the treatment of catatonia, particularly in refractory cases. While they are most used adjunctively, there are case reports describing their successful use as monotherapy [1]. Topiramate, in particular, has demonstrated effectiveness in patients unresponsive to standard treatments like lorazepam and valproate, supporting the hypothesis of glutamatergic hyperactivity in treatment-resistant catatonia. Its mechanism is multifaceted: it blocks state-dependent sodium channels, enhances GABA-A receptor activity by increasing chloride influx through a mechanism distinct from benzodiazepines and barbiturates, and antagonizes AMPA receptors, which are glutamatergic receptors co-localized with NMDA receptors. While it does not directly inhibit NMDA receptors, its AMPA antagonism may result in indirect NMDA modulation, contributing to its therapeutic effects [10].

The use of antipsychotics in catatonia remains one of the most controversial aspects of its management due to the risk of clinical worsening, conversion to malignant catatonia, and inducing NMS [1,12,14]. They should be used with extreme caution and avoided altogether in the absence of a primary psychotic disorder [12]. First-generation (typical) antipsychotics have higher rates of extrapyramidal side effects and NMS; thus, they are generally avoided in catatonic patients [1]. When antipsychotics are deemed necessary (typically in cases of catatonia associated with primary psychotic disorders such as schizophrenia), second-generation (atypical) antipsychotics are preferred due to their lower risk profile, and they should be administered in combination with a benzodiazepine to reduce complications [1,9]. Importantly, antipsychotics should never be used in patients currently displaying malignant catatonia or NMS, and caution is also warranted in delirious mania, where antipsychotics may exacerbate symptoms [1,19]. Before initiating antipsychotics, it is advisable to check serum iron levels, as low serum iron is a known risk factor for developing NMS [1].

Zolpidem, a non-benzodiazepine sedative-hypnotic and selective positive allosteric modulator of the α1 subunit of the GABA-A receptor, has shown potential in the treatment of catatonia, particularly in cases where first-line interventions like benzodiazepines or ECT are unavailable or ineffective [8,11-12]. Its unique pharmacodynamic profile, distinct from benzodiazepines, which act more broadly across multiple GABA-A subunits, may account for its clinical efficacy in catatonia, especially considering the suspected role of α1 subunit dysregulation in neuropsychiatric syndromes including schizophrenia, mood disorders, and autoimmune encephalitis [11]. In catatonia, dysfunction in the cortico-cortical circuit (particularly involving the primary motor cortex (PMC), supplementary motor area (SMA), and medial prefrontal cortex) has been implicated in impaired motor control and psychomotor symptoms. Reduced GABA-A receptor concentration in these areas is thought to result in decreased inhibition and disorganized motor output. Zolpidem’s selective action at the α1 subunit may help restore inhibitory tone in these regions via positive allosteric modulation, thereby normalizing motor function. Neuroimaging studies have demonstrated increased activity in GABAergic signaling pathways following zolpidem administration, supporting the hypothesis that its clinical effects in catatonia are mediated through restoration of cortical inhibitory control [8]. Zolpidem has been used in multiple therapeutic strategies: as an initial challenge agent, as monotherapy, in combination with benzodiazepines either initially or after failed benzodiazepine therapy, and as an augmentation agent [11]. In terms of dosing, higher-than-standard doses (e.g., up to 30-40 mg/day) have been associated with better outcomes and may be necessary to saturate the α1 subunit effectively [8,11]. This contrasts with cases of poor response where only 10 mg was used and mirrors the general need to escalate GABAergic agents beyond traditional anxiolytic and sleeping doses in catatonia [11]. The therapeutic response, like with lorazepam, is typically short-lived (lasting three to six hours due to zolpidem’s half-life of one to four hours), highlighting the need for frequent dosing or careful scheduling [12]. In our patient, high-dose zolpidem was again used as an adjunctive GABA-A agonist during both the index hospitalization and the prior episode, supporting its potential role in chronic, recurrent catatonia. Zolpidem’s utility in improving catatonia-related mutism is of particular interest, especially considering findings that mutism predicts poor response to lorazepam [12]. Moreover, its potential to improve patient engagement and reduce the need for polypharmacy, particularly in patients with comorbid conditions like dementia, makes it a compelling therapeutic option [8,11]. Despite its promise, zolpidem remains an underutilized agent in catatonia management. Current literature is limited to case reports, with no randomized controlled trials or head-to-head comparisons with lorazepam, and further research is needed to clarify optimal dosing, long-term safety, efficacy, and potential differences in response based on etiology [8,11-12].

Dopamine agonists have also been used, but due to the risk of worsening psychosis, their use is generally discouraged [1]. Bromocriptine, a medication with dopaminergic effects, has been shown in a review article to significantly accelerate clinical recovery when added to supportive care [18].

Although ketamine has been reported to induce catatonic symptoms in some cases, at least one report describes a dramatic improvement in catatonia following a slow intravenous injection of a sub-anesthetic dose. However, further research is needed before this approach can be recommended for clinical use [12].

When ECT is indicated but unavailable, neuromodulation techniques such as repetitive transcranial magnetic stimulation (rTMS) (particularly over the bilateral dorsolateral prefrontal cortex) and tDCS may serve as promising alternatives, with systematic reviews reporting generally positive outcomes and minimal adverse effects [12].

Vagus nerve stimulation (VNS) is an approved add-on treatment for resistant depression. The significant neurochemical, neuroanatomical, and theoretical overlap between VNS mechanisms and catatonia pathophysiology suggests that VNS could be a promising therapeutic strategy in refractory cases, warranting further research [3].

Complications

Early identification and intervention in catatonia are essential, as the condition can lead to serious medical complications primarily due to poor oral intake and prolonged immobility [1,8]. Dehydration resulting from reduced fluid consumption predisposes patients to electrolyte imbalances and acute kidney injury. For catatonia persisting beyond several weeks, vitamin levels should be assessed and repleted as necessary. Malnutrition may develop over time, contributing to muscle atrophy, gastrointestinal hypomotility, and micronutrient deficiencies, and in prolonged cases may necessitate enteral feeding, such as in the case of our patient [1]. The immobility characteristic of catatonia further increases the risk for life-threatening complications such as deep vein thrombosis, pulmonary embolism, pressure ulcers, muscle contractures, and infections (including UTI) [8]. Additionally, urinary retention is a potential complication of catatonia itself and may be exacerbated by antipsychotic medications [1,20]. Severe catatonia often impairs cognition and communication, resulting in loss of decisional capacity (DC). In patients with a history of recurrent catatonia, advance directives can be useful if completed during periods of intact DC. When patients lack DC, clinicians should work with the appropriate surrogate decision-maker, consider the patient’s previously stated preferences and values, and consult ethics services if needed (especially when proposing treatments like ECT). Advance directives, if available, may offer guidance on the patient's preferences regarding such interventions [1].

Limitations

This case report has several important limitations. First, as a single-case design, it cannot establish causality between UTI, systemic infection, and catatonic relapse; the temporal associations observed are hypothesis-generating rather than mechanistically conclusive. Second, diagnostic overlap between catatonia, delirium, and sepsis-associated encephalopathy cannot be fully excluded, as formal delirium assessment using structured instruments was not systematically documented. Third, multiple simultaneous interventions, including infection treatment, correction of metabolic derangements, benzodiazepines, zolpidem, memantine, and supportive ICU care, prevent attribution of clinical improvement to any single agent. Fourth, inflammatory biomarkers, CSF studies, autoimmune workup, and neuroimaging were not performed, limiting mechanistic interpretation.

## Conclusions

Catatonia remains a complex and understudied condition with multifactorial etiologies, and this case highlights the significant challenges of managing recurrent, treatment-resistant presentations in patients with substantial medical comorbidities and limited therapeutic options. Early recognition, structured diagnostic assessment, and thorough evaluation of potential medical contributors are essential, particularly when standard treatments fail. The incomplete understanding of the neurochemical, immunological, and autonomic mechanisms underlying catatonia further underscores the need for research aimed at refining diagnostic criteria, defining clinical phenotypes, clarifying the role of comorbidities, and evaluating both established and emerging therapies through rigorous clinical trials. Ultimately, catatonia illustrates the broader principle that psychiatric illness is medical illness, reinforcing the importance of equipping clinicians with the skills to recognize and treat it. Continued investigation in these areas may pave the way for more precise diagnostic approaches and individualized treatment strategies capable of improving outcomes in medically complicated or treatment-refractory catatonia. Recurrent or treatment-resistant catatonia in medically complex institutionalized patients should prompt systematic evaluation for infection, urinary retention/UTI, and metabolic derangements, as timely identification and treatment may be critical to psychiatric recovery.
